# Supplementary material for: 4‐Aminopyridine promotes functional recovery and remyelination in acute peripheral nerve injury
Source: EMBO Mol Med. 2016 Nov 14;8(12):1409–20. doi: 10.15252/emmm.201506035 (PMC5167128; doi:10.15252/emmm.201506035)
Supplement: Supplementary file 1 — Appendix [file EMMM-8-1409-s001.pdf]

Table of Contents

Materials and Methods
Reagents and Antibodies……..2
Mouse Model of Peripheral Nerve Injury……2
Fabrication of (4-AP)-PLGA Carriers……3
1. Water/Oil/Water Double Emulsion……3
2. Solvent Casting Technique……3
3. Rhodamine-Labeled PLGA Particles……3
4. PEG Hydrogel Polymerization……4
5. Analysis of 4-AP release kinetics ……4
Localized 4-AP application……..5
Biodegradation of PLGA carriers in vivo ……5
Sciatic Function Index (SFI) Determined by Walking Track Analysis ……6
Analysis of nerve conduction velocity……..7
Assessment of Neuropathic Pain……..7
Electronic von Frey Testing ……..8
Hargreaves testing ……..9
Treatment with 4-AP, tetraethylammonium (TEA), 3, 4-diaminopyridine (DAP) or neostigmine……..9
Transmission Electron Microscopy (TEM) ……10
Immunofluorescence analysis……10
Immunoblotting……..11
Image Analysis……..11
References……..12
Appendix Figures and Legends
Appendix Figure S1……..13
Appendix Figure S2……..14
Appendix Figure S3……..15

Materials and Methods

Reagents and Antibodies

4-Aminopyridine, Rhodamine-B, Poly (D,L-lactide-co-glycolide) (50:50, acid terminated, average Mw 38,000-54,000) were purchased from Sigma Aldrich. Anti-P_0 monoclonal antibodies were purchased from Aves Labs Inc.; anti-b-actin monoclonal antibodies were purchased from Santa Cruz Biotechnology; Hydrogel PEGDM was kindly provided by Danielle S.W. Benoit (University of Rochester).

Mouse Model of Peripheral Nerve Injury

All animal experiments described in this report were reviewed and approved by the University Committee on Animal Resources (IACUC) at the University of Rochester Medical Center. Ten-week-old female C57BL6 mice weighing 20 to 25 g were anesthetized with an intraperitoneal injection of ketamine (60 mg/kg) and xylazine (4 mg/kg). A lateral skin incision along the length of the femur was made and a direct lateral approach through the iliotibial band was performed to expose the sciatic nerve lying directly posterior to the femur. The sciatic nerve was bluntly exposed, and then the mice underwent one of the following randomly assigned procedures: (1) wound closure without manipulation of the nerve (sham-surgery group), (2) a sciatic nerve crush injury of thirty seconds duration (crush-injury group), as described in, e.g., (Magill et al., 2007, Elfar et al., 2008). Injury was created proximal to the tibial and peroneal divisions and was created with a smooth forceps with a metal ring around the forceps by pushing the ring from the tail-end to the head-end of the forceps to a constant position to assure applying the same amount of mechanical pressure to the sciatic nerve each time. Buprenorphine (0.05 mg/kg) was used for postoperative analgesia on the day of surgery and was given every twelve hours until the mice were able to walk without signs of pain.

Fabrication of (4-AP)-PLGA Carriers

1. Water/Oil/Water Double Emulsion

For the double emulsion technique, 6mg of 4-AP was dissolved in 0.3ml of deionized water and the drug solution was added to 3ml of 5% w/v PLGA in dichloromethane (DCM) and emulsified with a high speed homogenizer (Polytron) at 21,000rpm for 90sec to yield a w/o emulsion. Next, the water/oil (w/o) primary emulsion was added to 10ml PVA aqueous solution (2.5%) and further emulsified at 13,500rpm for 90sec. The organic solvent was allowed to evaporate by stirring the mixture at 1,000rpm for 18hrs under ambient temperature. The obtained microparticles were collected by centrifugation at 8,000rpm for 10min and washed twice with deionized water (Chaisri et al., 2009). The morphology of (4-AP)-PLGA films assessed by scanning electron microscopy (FIB- SEM, Zeiss Auriga). The samples were prepared by air-drying on a substrate and sputter- coating with gold (Denton Vacuum Desk II) before imaging.

2. Solvent Casting Technique

50mg of 50:50 PLGA polymer and 3mg of 4-AP were dissolved in 1mL of dichloromethane, and the solvent was cast on a glass coverslip or in a syringe. Samples were placed within a chemical hood with constant air flow at ambient temperature until the dichloromethane was completely evaporated. Thus, the amount of 4-AP provided was entirely enclosed within the PLGA bricks, and the theoretical encapsulating capacity was equal to the maximum solubility of 4-AP in dichloromethane. The morphology of (4-AP)-PLGA films was assessed by scanning electron microscopy (FIB- SEM, Zeiss Auriga). The samples were prepared by air-drying on a substrate and sputter- coating with gold (Denton Vacuum Desk II) before imaging.

3. Rhodamine-Labeled PLGA Particles

Using the same water/oil/water double emulsion mentioned above to make PLGA particles, 6mg of 4-AP was dissolved in 0.3ml of deionized water and the drug solution was added to 3ml of 5% w/v PLGA and 10mg of rhodamineB in dichloromethane (DCM) and emulsified with a high speed homogenizer (Polytron) at 21,000rpm for 90 sec to yield a w/o emulsion. Next, the w/o primary emulsion was added to 10ml PVA aqueous solution (2.5%) and further emulsified at 13,500rpm for 90sec. The organic solvent was allowed to evaporate by stirring the mixture at 1,000rpm for 18hrs under ambient temperature. The microparticles generated in this way were collected by centrifugation at 8,000rpm for 10min and washed twice with deionized water (Chaisri et al., 2009).

4. PEG Hydrogel Polymerization

5mg of (4-AP)-PLGA particles suspended with 20μL of PEG hydrogels consisting of 10 wt.% PEGDM with 0.05 wt.% lithium phenyl-2,4,6-trimethylbenzoylphosphinate (LAP) as a photoinitiator and were formed in a plastic tube mold (0.02 inches in diameter) via photopolymerization using long-wavelength 365 nm light (~5mW cm^2 intensity) for 10min (Hoffman et al., 2014).

5. Analysis of 4-AP release kinetics

To determine the amount of 4-AP contained in particles or films, 25mg of (4-AP)-PLGA particles or films were dissolved in 100μL dichloromethane (100μL) and then extracted in 1000μL of water. The amount of 4-AP in water was measured by spectrophotometry at 260nm with a series of 4-AP standards. The concentration was extrapolated based on the linear regression equation we got from the standards.

To determine the 4-AP releasing kinetics from the particles and films, 100mg of (4-AP)-PLGA particles or 40mg of (4-AP)-PLGA films were placed in 1000μL of PBS on a

vertical wheel rotator at room temperature. The amount of accumulative 4-AP released from the carriers into PBS was measured by spectrophotometer periodically, at time points as shown in Figure S1.

Localized 4-AP application

For localized application of 4-AP, 5mg of (4-AP)-PLGA particles (containing ~10µg 4-AP) were suspended in 20µL PEG hydrogel then photopolymerized in a plastic tube mold (0.02 inches in diameter) to form a (4-AP)-PLGA particles/PEG hydrogel ribbon. This ribbon was placed at the crushed site of the injured nerve immediately after the surgery. For (4-AP)-PLGA films, 5mg of film (containing 300µg 4-AP) was shredded to yield fragments of about 1mm x 3mm size, which were placed at the crushed site of the sciatic nerve in 20µL PEG hydrogel, followed by wound closure procedure. The dosages were selected to establish a lower effective thresholds for local 4-AP application, with particles containing the equivalent of less than a single day's total body dose of our lower systemic 4-AP dosage and films containing 6 times the upper systemic 4-AP daily dosage of 4-AP.

Biodegradation of PLGA carriers in vivo

To test the degradation rate and the special characteristics of locally delivered (4-AP)-PLGA carriers of the implantation in a specific location in vivo, PLGA particles were labeled with rhodamine to trace the remaining amount and the migration of PLGA carriers overtime in living animals. Rhodamine-labeled PLGA particles were implanted directly onto the crush-injured sciatic nerve of mice, and intensity change of fluorescence was monitored using In Vivo Imaging System (IVIS) to study the degradation rate of PLGA particles in vivo (Figure 3.3Ci). Fluorescence intensity fell over the two weeks after implantation (Figure 3.3Cii). However, during this period of time, the rhodamine-

labeled PLGA seemed to stay at a consistent location that we initially implanted (Figure 3.3Ci). Recovery surgery performed at the site three weeks after implantation revealed a small volume of PLGA carriers surrounding the sciatic nerve (Figure 3.3Ciii). This indicated that even though the carriers were still at the site of implantations the fluorescence intensity decreased quickly below the threshold to be detected through the muscle and skin tissues above the sciatic nerve two weeks after the implantation. However, this was one evidence that the fabricated PLGA carriers can stay at a consistent location and slowly degraded to release encapsulated drug over three weeks.

Sciatic Function Index (SFI) Determined by Walking Track Analysis

The assessment of motor function recovery was performed by calculating the sciatic function index (SFI). Walking track analysis was performed according to a published model that quantifies sciatic nerve function performance (de Medinaceli et al., 1982). Assessment of gait in this model has been extensively described previously (Elfar et al., 2008). Briefly, individual mouse footprints were obtained by painting each foot prior to mice walking a 50cm path down a narrow corridor lined with paper. Two observers who were blinded to the study protocol measured walking tracks only after randomization of all completed sample data to ensure proper blinding. Gait was measured from the metrics of resulting footprints: (1) toe spread (TS) (first through fifth toes), (2) total print length (PL), and (3) intermediate toe spread (ITS) (second, third, and fourth toes) of both limbs. When footprints were sufficiently abnormal as to make toe spread more difficult to discern by visual inspection of unmagnified images, measurements were made under magnification, so as not to remove footprints demonstrative of abnormal motor function from the analysis. All three measurements from 3 clearly inked randomly chosen footprints per trial were taken from the normal (N) and experimental (E) sides, and the SFI was calculated using the following formula: SFI = -38.3((EPL-NPL)/NPL) +

109.5((ETS-NTS)/NTS) + 13.3((EIT-NIT)/NIT) – 8.8, where E is the injured limb and N is the control limb as in previous studies (Gladman et al., 2012).

Analysis of nerve conduction velocity

Nerve conduction studies were performed by electrical stimulation of a nerve and recording the compound muscle action potential (CMAP) from needle electrodes overlying a muscle supplied by that nerve, as in, e.g., (Gupta and Steward, 2003, Osuchowski et al., 2009)). The EMG method was performed by means of subdermal stainless steel needle electrode placed into the hindlimbs (6V, 0.1ms, 1Hz, 5–15mA). The stimulating electrode was placed in resting muscle on gluteal fold to obtain the first CMAP. Then the stimulating electrode was moved to popliteal fossa with a 10mm fixed distance from gluteal fold to get the second record of CMAP. The nerve conduction velocity (NCV) was determined from the latencies of the potentials and the distance between two stimulating positions (10mm). The muscle was stimulated by a monopolar needle electrode, with a fixed frequency of 1Hz. Recording electrode was inserted in the tibialis anterior muscle approximately 3 mm above the heel. The reference recording electrode was inserted into the plantar aspect of the foot, and the reference stimulating electrode was inserted into the ipsilateral lumbar paraspinal muscles. All experiments, in this and other sections, were repeated at least three times, with a minimum of five (and usually eight) mice per treatment group.

Assessment of Neuropathic Pain.

The development of mechanical allodynia and thermal hyperalgesia were evaluated using the von Frey and Hargreaves Test, respectively. These assessments were performed on day 0 (pre-surgery) for a baseline, followed by post-operatively on days 3, 5, 8, 11, 14, and 21. Prior to each testing round, mice were placed on the respective

apparatus and allowed to habituate for 15 minutes. Two investigators performed each experiment with one controlling the nociceptive stimulus while the other recorded the values that elicited a response of the hindpaw associated with discomfort (hind paw retraction, licking, shaking, flinching, four-paw jumping following stimulation). The hand-held unit displaying this value was visible to the recorder only, to ensure proper blinding.

Electronic von Frey Testing: Mechanical allodynia was evaluated by the von Frey test (Kiguchi et al., 2010a, Kiguchi et al., 2010b) using Electronic von Frey (EvF) Anesthesiometer (IITC Life Science, Woodland Hills, CA) following the method previously described (Cunha et al., 2004, Martinov et al., 2013). Mice were placed in clear plexiglass boxes (7 cm x 9 cm x 7 cm) on an elevated wire mesh screen, with a total of four mice on the apparatus at one time. A rigid polypropylene tip (diameter = 0.8 mm) from the Semmes-Weinstein monofilament set was secured to the 90-gram pressure probe, and pressure was then applied to the plantar surface of each hindpaw with increasing force until a withdrawal response was observed. The maximum force value of pressure (in grams) that caused paw withdrawal was considered to be the mechanical threshold (g) and was recorded. The left paw of all four mice were probed first one after the other, followed by the contralateral right paw so that all mice hindpaws were assessed once with the withdrawal thresholds recorded for each paw. This procedure was then repeated in the same way four more times so that each mouse had 5 withdrawal threshold values measured per paw at each time point. The 5 values were averaged to give each mouse one daily value per hindpaw. These values were averaged and data are expressed per treatment group as the mean ± SEM at each time point (prior to injury, and every other day post-injury).

Hargreaves testing: Thermal hyperalgesia was assessed by the Hargreaves test (Hargreaves et al., 1988) using the Plantar Test Analgesia Meter, Hargreaves Apparatus (IITC Life Science, Woodlands, CA). The mice were placed in a Plexiglass chamber on top of a tempered glass floor and allowed to acclimatize, as described earlier. A focused radiant heat light source with constant intensity (set to an active intensity (AI) of 39) was focused on the central plantar surface of the hind paw using the Hargreaves machine. A withdrawal response shuts off the light source automatically, and the paw withdrawal latency, defined as the time (in seconds) from initial heat exposure to the withdrawal response, was recorded. A cut off time for this test was preset for 15 s to avoid tissue damage, and each hindpaw had 5-minute intervals between trials to prevent thermal sensitization. A total of three trials each were collected from the left and right hind paws of each mouse. Data are represented as means +/- SEM for withdrawal latency (s).

Treatment with 4-AP, tetraethylammonium (TEA), 3, 4-diaminopyridine (DAP) or neostigmine

All treatments were initiated 24 hrs after injury. In the case of examination of transient improvements in outcome that might be useful for diagnostic purposes, measurements were conducted 1 hr after intraperitoneal injection of either 4-AP or neostigmine. Neostigmine treatment used a dosage of 0.7 mg/kg, the pediatric dose for reversing nerve blockade (as extrapolated from (Barnes and Eltherington, 1973).

In the case of examination of durable changes, measurements were conducted ~22 hrs after treatment, a time when all drug levels will have fallen below physiologically effective levels. DAP was applied at the same dose as 4AP, while TEA was applied at the higher dose of 5mg/kg (based on (Barnes and Eltherington, 1973), this being a dose high enough to cause cardiovascular effects in dogs (which have an almost identical LD_{50} as

mice), thus pushing dosage levels to acceptable limits). This is only modestly above the dose of TEA (2mg/kg) reported to provide benefit in a 6-hydroxydopamine model of Parkinson's disease (Haghdoost-Yazdi et al., 2011), and thus is within the concentration in mice known to be biologically active.

Transmission Electron Microscopy (TEM)

The sciatic nerves were immersion fixed overnight at 4^oC in 2.5% glutaraldehyde and 4.0% paraformaldehyde in 0.1M sodium cacodylate buffer. The nerves were rinsed in 0.1M sodium cacodylate buffer and post-fixed for one hour in 1.0% osmium tetroxide combined with 1.0% potassium ferrocyanide. After rinsing in distilled water, the sections were dehydrated in a graded series of ethanol to 100% 3 times, transitioned into propylene oxide followed by Epon/Araldite epoxy resin overnight and finally embedded and polymerized at 60^oC for 48 hours. Using an ultramicrotome and a diamond knife, thin-sections (70nm) were collected onto 150 mesh nickel grids and stained with uranyl acetate and lead citrate. The stained grids were examined using a Hitachi 7650 TEM and photographed using an attached Gatan Erlangshen 11 megapixel digital camera system.

Immunofluorescence analysis

The experimental and contralateral (uninjured) sciatic nerves from each test group were harvested at specific time points during healing and recovery. The nerves were bluntly dissected from the dorsal root ganglion to a point distal to the peroneal-tibial nerve divisions. All nerves were fixed in 4% paraformaldehyde solution for three hours and embedded in paraffin to evaluate cross sections. Slides were pretreated with 0.01M citrate buffer (pH 6.0) for antigen retrieval. Nonspecific blocking was performed with 1:20 diluted serum for 30min. Sequentially sectioned slides were incubated with a

primary antibody overnight, followed by incubation with a fluorescent-labeled secondary antibody for 1hr.

Immunoblotting

The crushed site of the sciatic nerve at 21 days post-injury was collected and lysed in cell extraction buffer (Invitrogen). The 2mm length of crushed sciatic nerve was placed in a 1.5mL microtube with 100μL cell extraction buffer. The tissue was frozen in liquid nitrogen and thawed repetitively for three times. After that, the tissue was grinded by pestle till no chunks could be visualized. Samples were resolved on SDS-PAGE gels and transferred to PVDF membranes (PerkinElmer Life Science, Wellesley, MA, USA). After being blocked in 5% bovine serum albumin in PBS containing 0.1% Tween 20, membranes were incubated with a primary antibody, followed by incubation with a HRP-conjugated secondary antibody (Santa Cruz Biotechnology). Membranes were visualized using Western Blotting Luminol Reagent (Santa Cruz Biotechnology) and imaging system.

Image Analysis

Images of cross-sectioned sciatic nerve taken by TEM were processed by ImageJ (US National Institutes of Health, Bethesda, Maryland, USA) to determine myelin area, myelin thickness, and g^{area}-ratio on myelinated axonal fibers. Axonal area, axonal circularity, and the number of myelinated and total axon were also counted. For analyzing myelin thickness, 15 randomly chosen myelinated axons were analyzed for each mouse, and 6 thicknesses of myelin sheath (at equal degrees of separation around a central point) were measured on each axon. For determining the axonal area, myelin area and g^{area}-ratio: 40 randomly chosen axons were analyzed in each mouse. Immunofluorescence images of P_0 expression in cross-sectioned sciatic nerve were

analyzed by ImageJ to determine the average fluorescence intensity of axons-associated P_0 labeling.

References

Barnes LG, Eltherington CD (1973) Dug Dosage in Laboratory Animals: A Handbook. California: University of California Press.

Chaisri W, Hennink WE, Okonogi S (2009) Preparation and characterization of cephalexin loaded PLGA microspheres. Current drug delivery 6:69-75.

Cunha TM, Verri WA, Jr., Vivancos GG, Moreira IF, Reis S, Parada CA, Cunha FQ, Ferreira SH (2004) An electronic pressure-meter nociception paw test for mice. Brazilian journal of medical and biological research = Revista brasileira de pesquisas medicas e biologicas / Sociedade Brasileira de Biofisica [et al] 37:401-407.

de Medinaceli L, Freed WJ, Wyatt RJ (1982) An index of the functional condition of rat sciatic nerve based on measurements made from walking tracks. Exp Neurol 77:634-643.

Elfar JC, Jacobson JA, Puzas JE, Rosier RN, Zuscik MJ (2008) Erythropoietin accelerates functional recovery after peripheral nerve injury. J Bone Joint Surg Am 90:1644-1653.

Gladman SJ, Huang W, Lim SN, Dyall SC, Boddy S, Kang JX, Knight MM, Priestley JV, Michael-Titus AT (2012) Improved outcome after peripheral nerve injury in mice with increased levels of endogenous omega-3 polyunsaturated fatty acids. J Neurosci 32:563-571.

Gupta R, Steward O (2003) Chronic nerve compression induces concurrent apoptosis and proliferation of Schwann cells. J Comp Neurol 461:174-186.

Haghdoost-Yazdi H, Faraji A, Fraidouni N, Movahedi M, Hadibeygi E, Vaezi F (2011) Significant effects of 4-aminopyridine and tetraethylammonium in the treatment of 6-hydroxydopamine-induced Parkinson's disease. Behavioural brain research 223:70-74.

Hargreaves K, Dubner R, Brown F, Flores C, Joris J (1988) A new and sensitive method for measuring thermal nociception in cutaneous hyperalgesia. Pain 32:77-88.

Hoffman MD, Van Hove AH, Benoit DS (2014) Degradable hydrogels for spatiotemporal control of mesenchymal stem cells localized at decellularized bone allografts. Acta biomaterialia 10:3431-3441.

Kiguchi N, Kobayashi Y, Maeda T, Saika F, Kishioka S (2010a) CC-chemokine MIP-1alpha in the spinal cord contributes to nerve injury-induced neuropathic pain. Neurosci Lett 484:17-21.

Kiguchi N, Maeda T, Kobayashi Y, Fukazawa Y, Kishioka S (2010b) Macrophage inflammatory protein-1alpha mediates the development of neuropathic pain following peripheral nerve injury through interleukin-1beta up-regulation. Pain 149:305-315.

Magill CK, Tong A, Kawamura D, Hayashi A, Hunter DA, Parsadanian A, Mackinnon SE, Myckatyn TM (2007) Reinnervation of the tibialis anterior following sciatic nerve crush injury: a confocal microscopic study in transgenic mice. Exp Neurol 207:64-74.

Martinov T, Mack M, Sykes A, Chatterjea D (2013) Measuring changes in tactile sensitivity in the hind paw of mice using an electronic von Frey apparatus. Journal of visualized experiments : JoVE e51212.

Osuchowski MF, Teener J, Remick D (2009) Noninvasive model of sciatic nerve conduction in healthy and septic mice: reliability and normative data. Muscle & nerve 40:610-616.

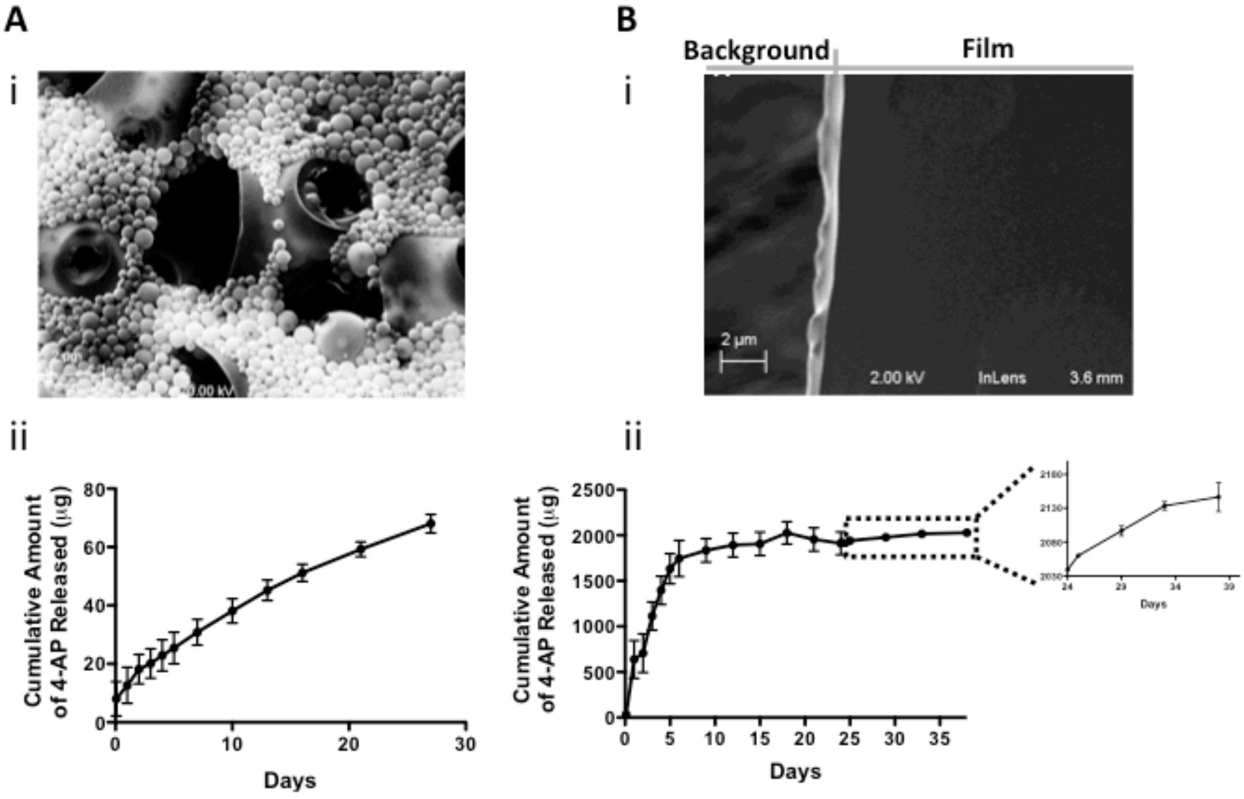

Appendix Figure S1. Physical characteristics of (4-AP)-PLGA carriers of encapsulated 4-AP. (A) (i) (4-AP)-PLGA microparticles made by a water/oil/water double emulsion technique displayed 200~500nm particle diameters. (ii) Measurement of cumulative amount of released 4-AP in vitro showed that 100mg of (4-AP)-PLGA particles continuously and steadily released 4-AP into PBS for at least 27 days. (B) (i) (4-AP)-PLGA films were made by solvent casting techniques. The PLGA film appears to be a flat, smooth, and evenly thin material with approximate 30-40μm thickness. (ii) Measurements of the cumulative amount of released 4-AP in vitro showed that 40mg of (4-AP)-PLGA films released 70% of encapsulated 4-AP at the first 7 days, but that the film continued to release additional 4-AP for approximately 38 days.

Appendix Figure S1

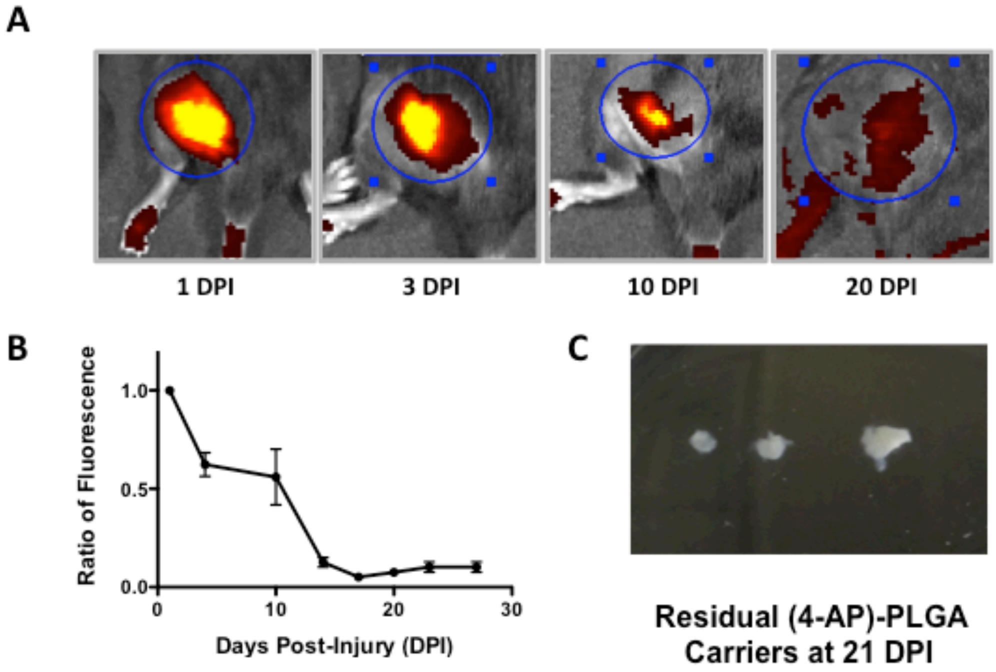

Appendix Figure S2. Rhodamine-labeled (4-AP)-PLGA carriers remained at the site of placement for at least 21 days. (A, B) Fluorescence emitted from the rhodamine-labeled PLGA carriers could be detected for 2 weeks after implantation. (C) With retrieval surgery, PLGA carriers were found at the site of the sciatic nerve crush injury at 21 days post-injury.

Appendix Figure S2

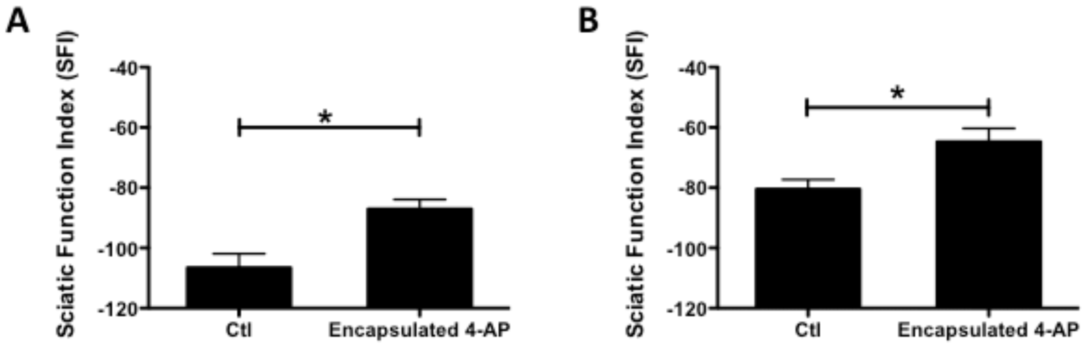

Appendix Figure S3. Bioactivity of encapsulated 4-AP within the (4-AP)-PLGA carriers .(A) Released 4-AP from (4-AP)-PLGA particles was collected and quantified, and then 50μg was administered to mice with sciatic nerve crush injuries at 5 days post-injury to test the bioactivity of encapsulated 4-AP. Significant SFI improvement was observed after 4-AP administration. (B) Released 4-AP from (4-AP)-PLGA films was collected and quantified, and then 50μg was administered to mice with sciatic nerve crush injuries quantified to test the bioactivity of the encapsulated 4-AP. Significant SFI improvement was observed immediately. (* i:p<0.05; two-tailed unpaired t-test; N=3 for each group)

Appendix Figure S3
