# Supplementary material for: 4‐Aminopyridine promotes functional recovery and remyelination in acute peripheral nerve injury
Source: EMBO Mol Med. 2016 Nov 14;8(12):1409–20. doi: 10.15252/emmm.201506035 (PMC5167128; doi:10.15252/emmm.201506035)
Supplement: Supplementary file 2 — Expanded View Figures PDF [file EMMM-8-1409-s002.pdf]

Expanded View Figures

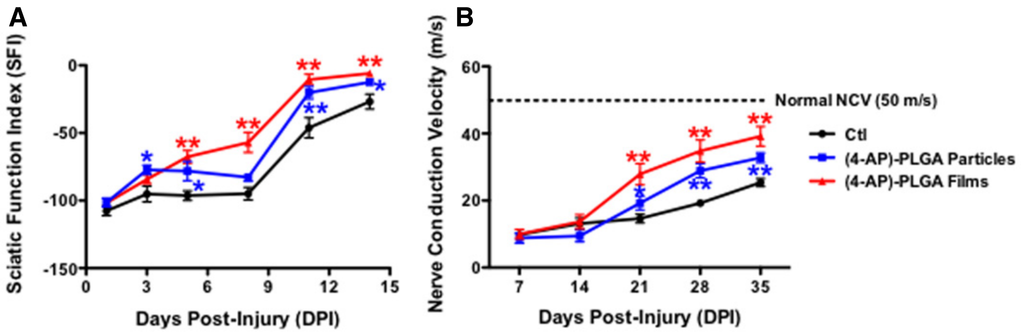

Figure EV1. Local administration of 4-AP in PLGA particles or films enhances functional and electrophysiological recovery after sciatic nerve crush.

A Local 4-AP-treated crushed sciatic nerve (black, vehicle PLGA films; red, (4-AP)-PLGA films; blue, (4-AP)-PLGA particles) regained partial walking ability as early as 3 days post-injury compared to vehicle-treated group (*$P < 0.05$; **$P < 0.01$; ANOVA with *post hoc* comparisons using two-tailed unpaired *t*-test; $N = 6$ for each group).

B Local 4-AP-treated crushed sciatic nerve (black, vehicle PLGA films; red, (4-AP)-PLGA films; blue, (4-AP)-PLGA particles) showed faster improvement in nerve conduction velocity restoration compared with vehicle-treated mice, beginning at 21 days post-injury. In addition, mice treated with the higher dosage 4-AP-containing films showed greater improvement than mice treated with the lower dosage of local 4-AP-containing particles (*$P < 0.05$; **$P < 0.01$; ANOVA with *post hoc* comparison using two-tailed unpaired *t*-test: $n = 6$ for each group).

Data information: Data are presented as mean ± SEM and show a representative experiment from 2 to 3 repetitions.

© 2016 The Authors
